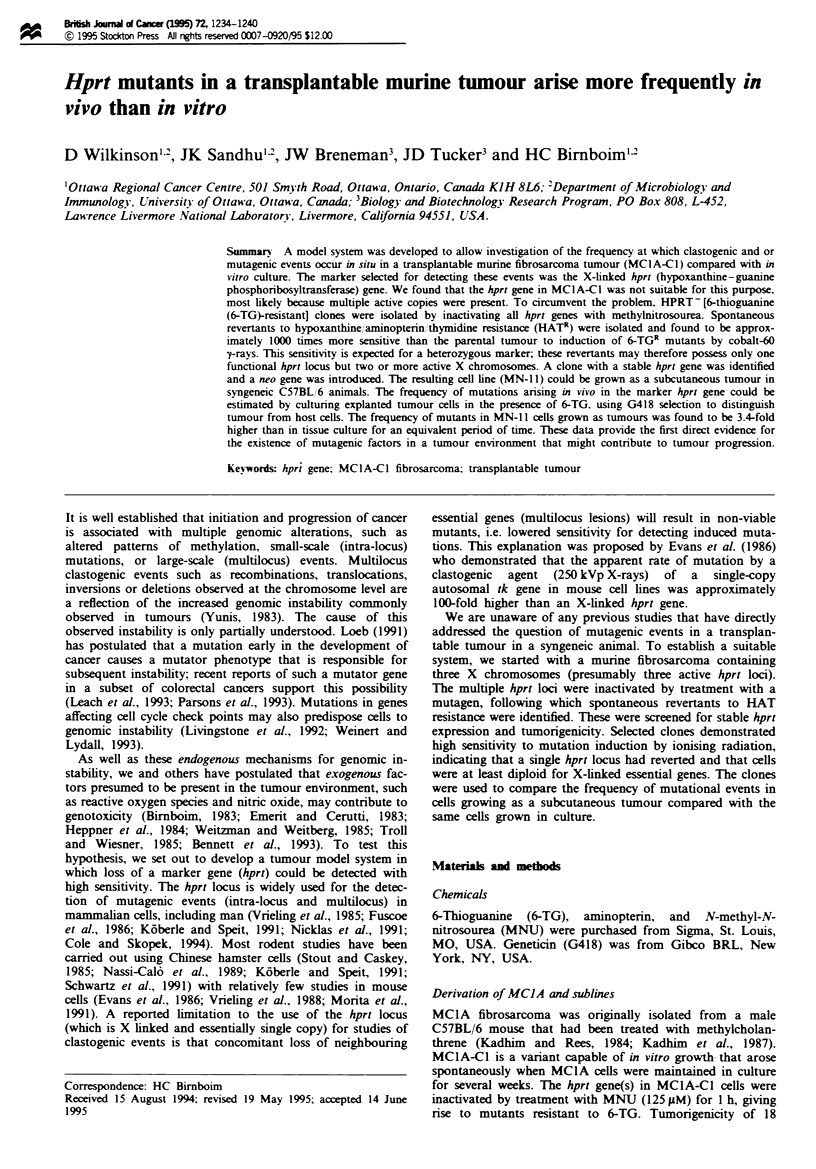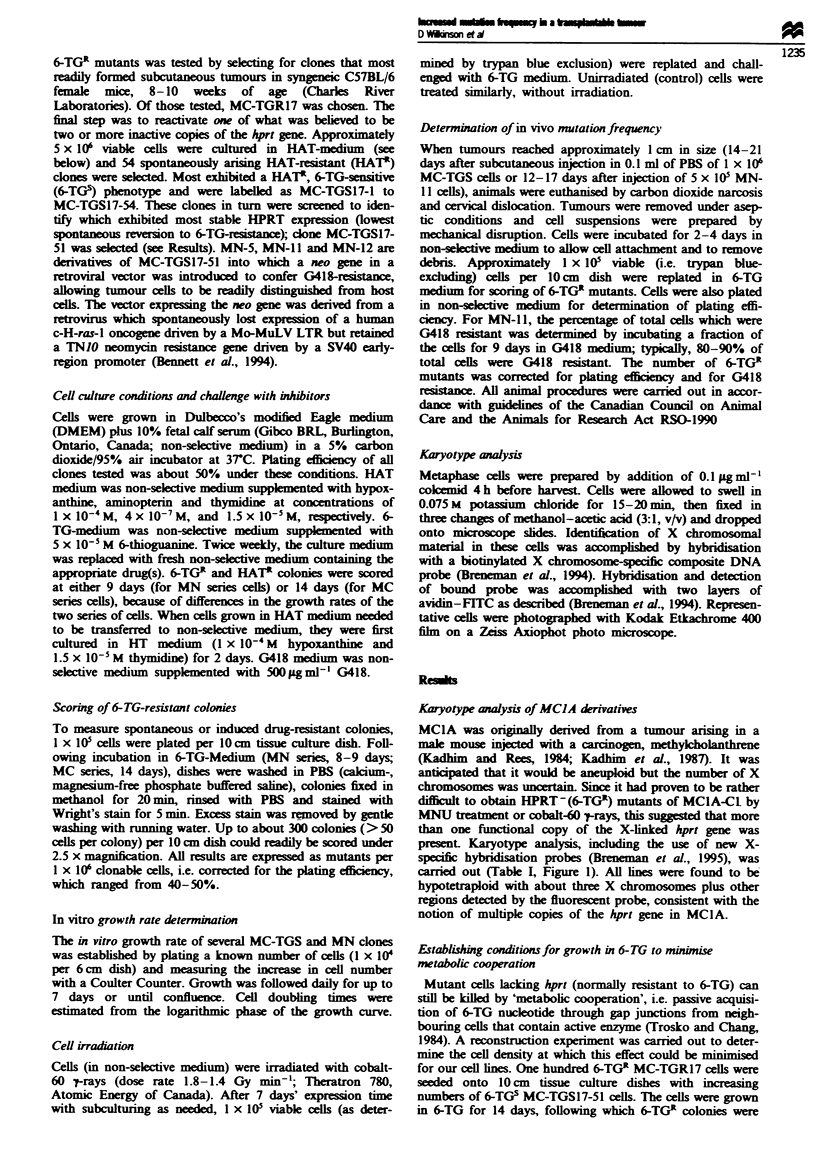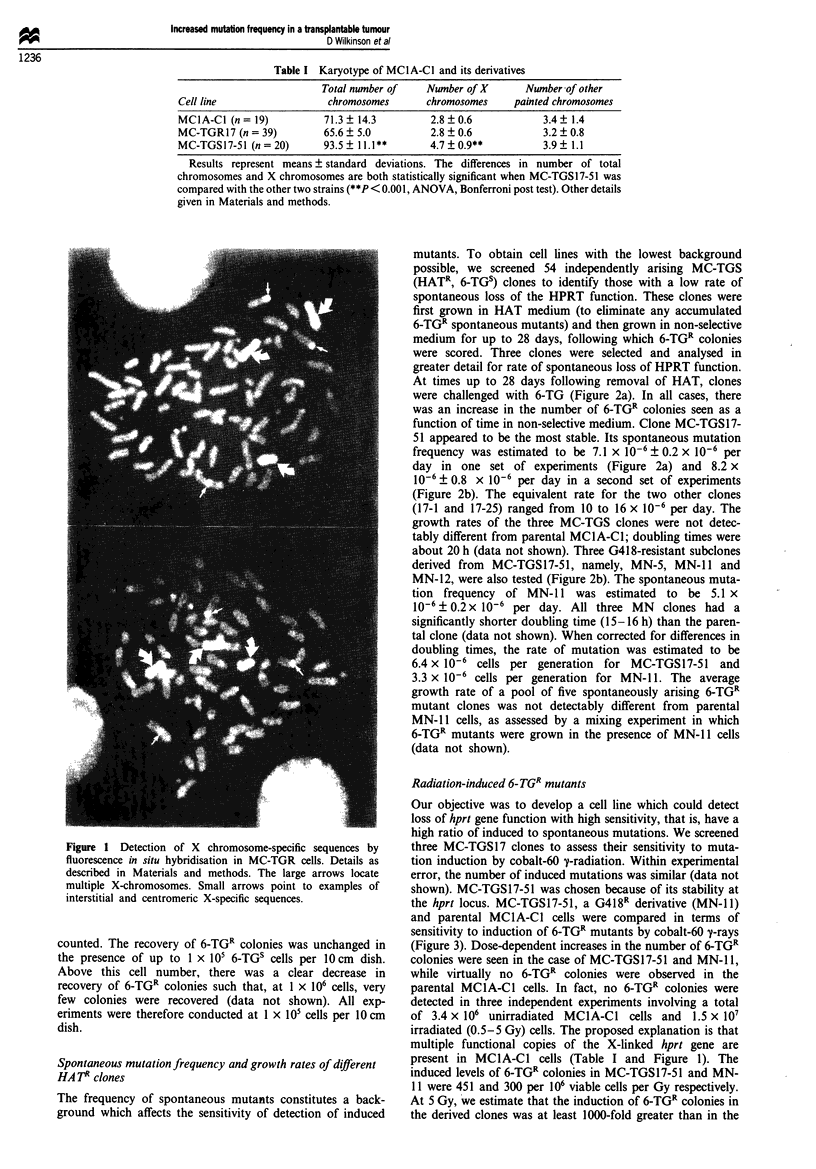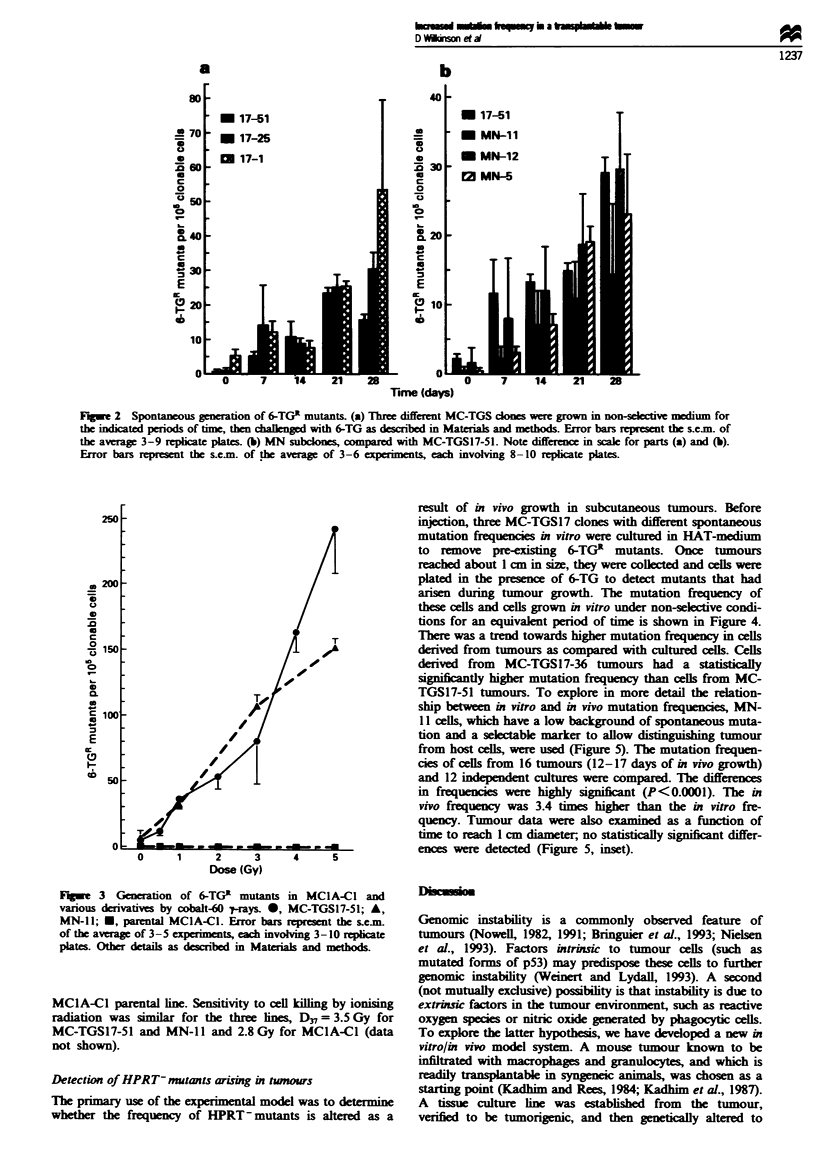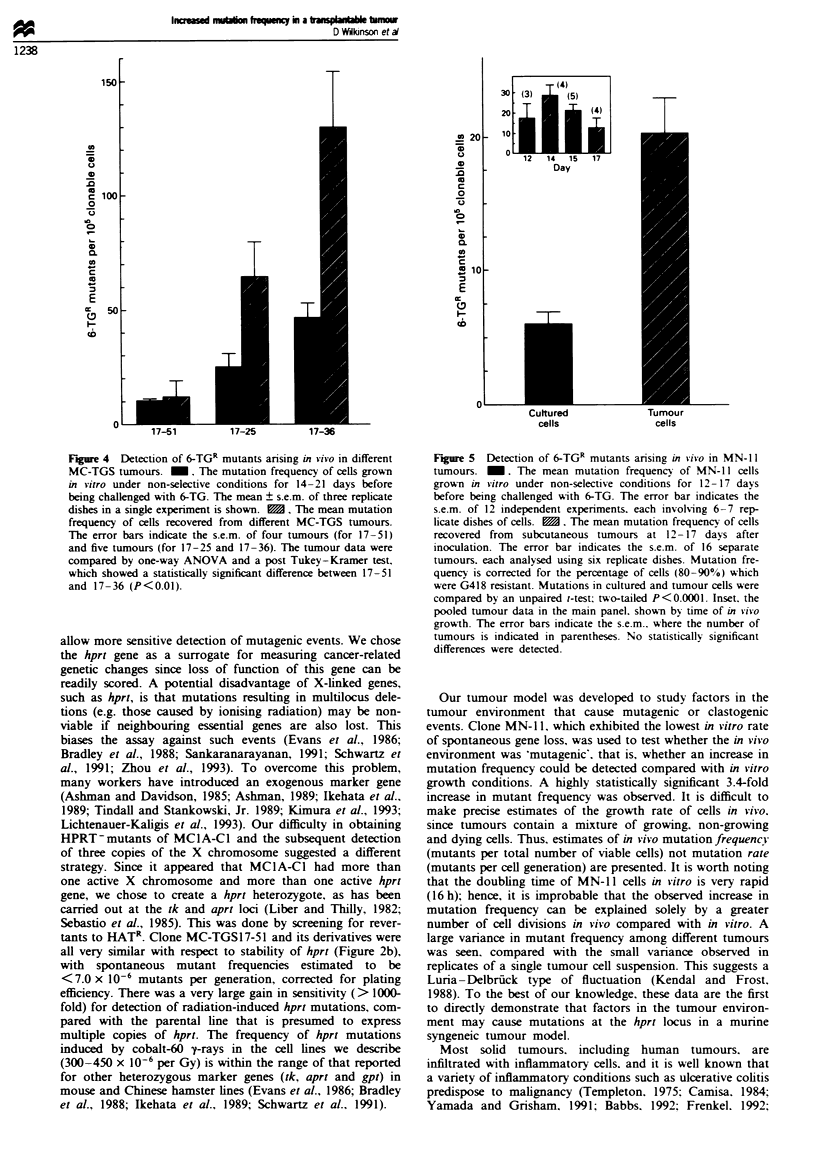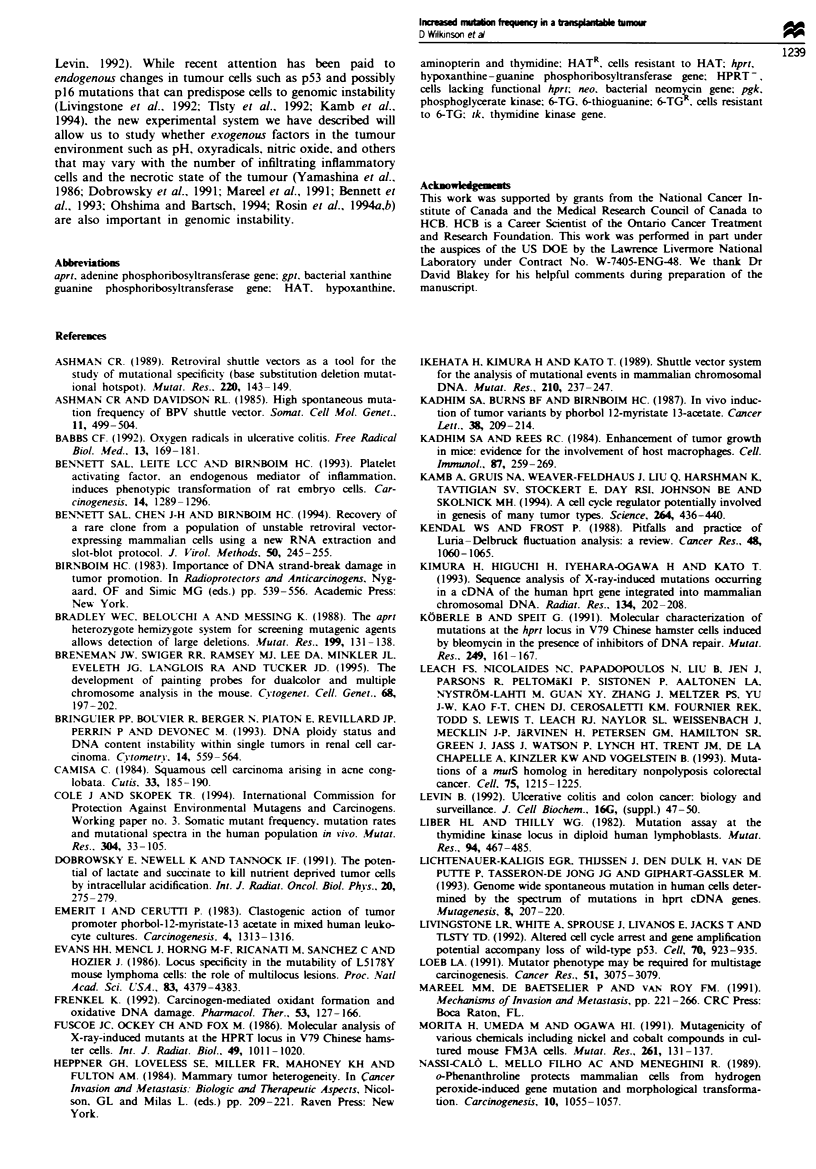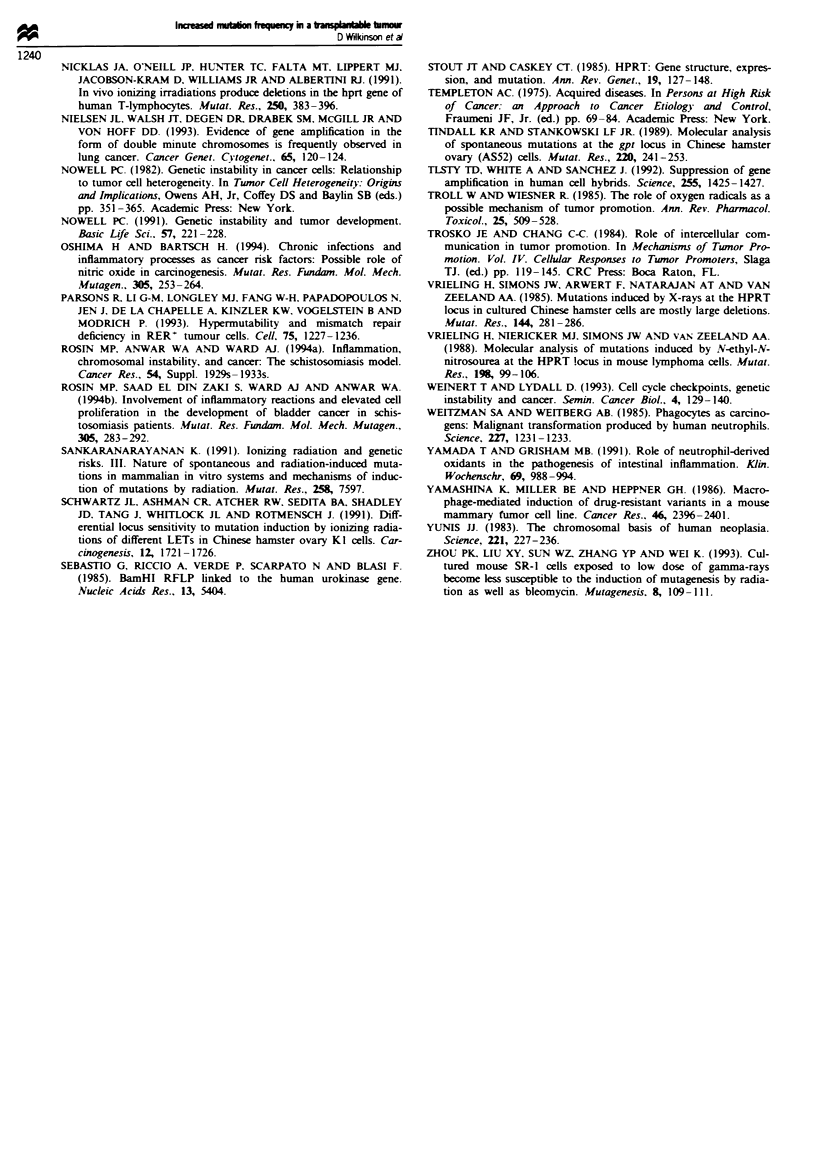# Hprt mutants in a transplantable murine tumour arise more frequently in vivo than in vitro.

**DOI:** 10.1038/bjc.1995.492

**Published:** 1995-11

**Authors:** D. Wilkinson, J. K. Sandhu, J. W. Breneman, J. D. Tucker, H. C. Birnboim

**Affiliations:** Ottawa Regional Cancer Centre, Ontario, Canada.

## Abstract

**Images:**


British Journal of Cancer (1995) 72, 1234–1240

© 1995 Stockton Press All rights reserved 0007-0920/95 $12.00

### *Hprt* mutants in a transplantable murine tumour arise more frequently *in vivo* than *in vitro*

D Wilkinson[1,2], JK Sandhu[1,2], JW Breneman[3], JD Tucker[3] and HC Birnboim[1,2]

[1]*Ottawa Regional Cancer Centre, 501 Smyth Road, Ottawa, Ontario, Canada K1H 8L6;* [2]*Department of Microbiology and Immunology, University of Ottawa, Ottawa, Canada;* [3]*Biology and Biotechnology Research Program, PO Box 808, L-452, Lawrence Livermore National Laboratory, Livermore, California 94551, USA.*

**Summary** A model system was developed to allow investigation of the frequency at which clastogenic and/or mutagenic events occur *in situ* in a transplantable murine fibrosarcoma tumour (MC1A-C1) compared with *in vitro* culture. The marker selected for detecting these events was the X-linked *hprt* (hypoxanthine–guanine phosphoribosyltransferase) gene. We found that the *hprt* gene in MC1A-C1 was not suitable for this purpose, most likely because multiple active copies were present. To circumvent the problem, HPRT$^-$ [6-thioguanine (6-TG)-resistant] clones were isolated by inactivating all *hprt* genes with methylnitrosourea. Spontaneous revertants to hypoxanthine/aminopterin/thymidine resistance (HAT$^R$) were isolated and found to be approximately 1000 times more sensitive than the parental tumour to induction of 6-TG$^R$ mutants by cobalt-60 γ-rays. This sensitivity is expected for a heterozygous marker; these revertants may therefore possess only one functional *hprt* locus but two or more active X chromosomes. A clone with a stable *hprt* gene was identified and a *neo* gene was introduced. The resulting cell line (MN-11) could be grown as a subcutaneous tumour in syngeneic C57BL/6 animals. The frequency of mutations arising *in vivo* in the marker *hprt* gene could be estimated by culturing explanted tumour cells in the presence of 6-TG, using G418 selection to distinguish tumour from host cells. The frequency of mutants in MN-11 cells grown as tumours was found to be 3.4-fold higher than in tissue culture for an equivalent period of time. These data provide the first direct evidence for the existence of mutagenic factors in a tumour environment that might contribute to tumour progression.

**Keywords:** *hprt* gene; MC1A-C1 fibrosarcoma; transplantable tumour

It is well established that initiation and progression of cancer is associated with multiple genomic alterations, such as altered patterns of methylation, small-scale (intra-locus) mutations, or large-scale (multilocus) events. Multilocus clastogenic events such as recombinations, translocations, inversions or deletions observed at the chromosome level are a reflection of the increased genomic instability commonly observed in tumours (Yunis, 1983). The cause of this observed instability is only partially understood. Loeb (1991) has postulated that a mutation early in the development of cancer causes a mutator phenotype that is responsible for subsequent instability; recent reports of such a mutator gene in a subset of colorectal cancers support this possibility (Leach *et al.*, 1993; Parsons *et al.*, 1993). Mutations in genes affecting cell cycle check points may also predispose cells to genomic instability (Livingstone *et al.*, 1992; Weinert and Lydall, 1993).

As well as these *endogenous* mechanisms for genomic instability, we and others have postulated that *exogenous* factors presumed to be present in the tumour environment, such as reactive oxygen species and nitric oxide, may contribute to genotoxicity (Birnboim, 1983; Emerit and Cerutti, 1983; Heppner *et al.*, 1984; Weitzman and Weitberg, 1985; Troll and Wiesner, 1985; Bennett *et al.*, 1993). To test this hypothesis, we set out to develop a tumour model system in which loss of a marker gene (*hprt*) could be detected with high sensitivity. The *hprt* locus is widely used for the detection of mutagenic events (intra-locus and multilocus) in mammalian cells, including man (Vrieling *et al.*, 1985; Fuscoe *et al.*, 1986; Köberle and Speit, 1991; Nicklas *et al.*, 1991; Cole and Skopek, 1994). Most rodent studies have been carried out using Chinese hamster cells (Stout and Caskey, 1985; Nassi-Calò *et al.*, 1989; Köberle and Speit, 1991; Schwartz *et al.*, 1991) with relatively few studies in mouse cells (Evans *et al.*, 1986; Vrieling *et al.*, 1988; Morita *et al.*, 1991). A reported limitation to the use of the *hprt* locus (which is X linked and essentially single copy) for studies of clastogenic events is that concomitant loss of neighbouring essential genes (multilocus lesions) will result in non-viable mutants, i.e. lowered sensitivity for detecting induced mutations. This explanation was proposed by Evans *et al.* (1986) who demonstrated that the apparent rate of mutation by a clastogenic agent (250 kVp X-rays) of a single-copy autosomal *tk* gene in mouse cell lines was approximately 100-fold higher than an X-linked *hprt* gene.

We are unaware of any previous studies that have directly addressed the question of mutagenic events in a transplantable tumour in a syngeneic animal. To establish a suitable system, we started with a murine fibrosarcoma containing three X chromosomes (presumably three active *hprt* loci). The multiple *hprt* loci were inactivated by treatment with a mutagen, following which spontaneous revertants to HAT resistance were identified. These were screened for stable *hprt* expression and tumorigenicity. Selected clones demonstrated high sensitivity to mutation induction by ionising radiation, indicating that a single *hprt* locus had reverted and that cells were at least diploid for X-linked essential genes. The clones were used to compare the frequency of mutational events in cells growing as a subcutaneous tumour compared with the same cells grown in culture.

### Materials and methods

### *Chemicals*

6-Thioguanine (6-TG), aminopterin, and *N*-methyl-*N*-nitrosourea (MNU) were purchased from Sigma, St. Louis, MO, USA. Geneticin (G418) was from Gibco BRL, New York, NY, USA.

### *Derivation of MC1A and sublines*

MC1A fibrosarcoma was originally isolated from a male C57BL/6 mouse that had been treated with methylcholanthrene (Kadhim and Rees, 1984; Kadhim *et al.*, 1987). MC1A-C1 is a variant capable of *in vitro* growth that arose spontaneously when MC1A cells were maintained in culture for several weeks. The *hprt* gene(s) in MC1A-C1 cells were inactivated by treatment with MNU (125 μM) for 1 h, giving rise to mutants resistant to 6-TG. Tumorigenicity of 18

Correspondence: HC Birnboim
Received 15 August 1994; revised 19 May 1995; accepted 14 June 1995

6-TG$^R$ mutants was tested by selecting for clones that most readily formed subcutaneous tumours in syngeneic C57BL/6 female mice, 8–10 weeks of age (Charles River Laboratories). Of those tested, MC-TGR17 was chosen. The final step was to reactivate *one* of what was believed to be two or more inactive copies of the *hprt* gene. Approximately $5 \times 10^6$ viable cells were cultured in HAT-medium (see below) and 54 spontaneously arising HAT-resistant (HAT$^R$) clones were selected. Most exhibited a HAT$^R$, 6-TG-sensitive (6-TG$^S$) phenotype and were labelled as MC-TGS17-1 to MC-TGS17-54. These clones in turn were screened to identify which exhibited most stable HPRT expression (lowest spontaneous reversion to 6-TG-resistance); clone MC-TGS17-51 was selected (see Results). MN-5, MN-11 and MN-12 are derivatives of MC-TGS17-51 into which a *neo* gene in a retroviral vector was introduced to confer G418-resistance, allowing tumour cells to be readily distinguished from host cells. The vector expressing the *neo* gene was derived from a retrovirus which spontaneously lost expression of a human c-H-*ras*-1 oncogene driven by a Mo-MuLV LTR but retained a TN*10* neomycin resistance gene driven by a SV40 early-region promoter (Bennett *et al.*, 1994).

### *Cell culture conditions and challenge with inhibitors*

Cells were grown in Dulbecco's modified Eagle medium (DMEM) plus 10% fetal calf serum (Gibco BRL, Burlington, Ontario, Canada; non-selective medium) in a 5% carbon dioxide/95% air incubator at 37°C. Plating efficiency of all clones tested was about 50% under these conditions. HAT medium was non-selective medium supplemented with hypoxanthine, aminopterin and thymidine at concentrations of $1 \times 10^{-4}$ M, $4 \times 10^{-7}$ M, and $1.5 \times 10^{-5}$ M, respectively. 6-TG-medium was non-selective medium supplemented with $5 \times 10^{-5}$ M 6-thioguanine. Twice weekly, the culture medium was replaced with fresh non-selective medium containing the appropriate drug(s). 6-TG$^R$ and HAT$^R$ colonies were scored at either 9 days (for MN series cells) or 14 days (for MC series cells), because of differences in the growth rates of the two series of cells. When cells grown in HAT medium needed to be transferred to non-selective medium, they were first cultured in HT medium ($1 \times 10^{-4}$ M hypoxanthine and $1.5 \times 10^{-5}$ M thymidine) for 2 days. G418 medium was non-selective medium supplemented with 500 $\mu$g ml$^{-1}$ G418.

### *Scoring of 6-TG-resistant colonies*

To measure spontaneous or induced drug-resistant colonies, $1 \times 10^5$ cells were plated per 10 cm tissue culture dish. Following incubation in 6-TG-Medium (MN series, 8–9 days; MC series, 14 days), dishes were washed in PBS (calcium-, magnesium-free phosphate buffered saline), colonies fixed in methanol for 20 min, rinsed with PBS and stained with Wright's stain for 5 min. Excess stain was removed by gentle washing with running water. Up to about 300 colonies (>50 cells per colony) per 10 cm dish could readily be scored under 2.5 × magnification. All results are expressed as mutants per $1 \times 10^6$ clonable cells, i.e. corrected for the plating efficiency, which ranged from 40–50%.

### In vitro *growth rate determination*

The *in vitro* growth rate of several MC-TGS and MN clones was established by plating a known number of cells ($1 \times 10^4$ per 6 cm dish) and measuring the increase in cell number with a Coulter Counter. Growth was followed daily for up to 7 days or until confluence. Cell doubling times were estimated from the logarithmic phase of the growth curve.

### *Cell irradiation*

Cells (in non-selective medium) were irradiated with cobalt-60 $\gamma$-rays (dose rate 1.8–1.4 Gy min$^{-1}$; Theratron 780, Atomic Energy of Canada). After 7 days' expression time with subculturing as needed, $1 \times 10^5$ viable cells (as determined by trypan blue exclusion) were replated and challenged with 6-TG medium. Unirradiated (control) cells were treated similarly, without irradiation.

### *Determination of* in vivo *mutation frequency*

When tumours reached approximately 1 cm in size (14–21 days after subcutaneous injection in 0.1 ml of PBS of $1 \times 10^6$ MC-TGS cells or 12–17 days after injection of $5 \times 10^5$ MN-11 cells), animals were euthanised by carbon dioxide narcosis and cervical dislocation. Tumours were removed under aseptic conditions and cell suspensions were prepared by mechanical disruption. Cells were incubated for 2–4 days in non-selective medium to allow cell attachment and to remove debris. Approximately $1 \times 10^5$ viable (i.e. trypan blue-excluding) cells per 10 cm dish were replated in 6-TG medium for scoring of 6-TG$^R$ mutants. Cells were also plated in non-selective medium for determination of plating efficiency. For MN-11, the percentage of total cells which were G418 resistant was determined by incubating a fraction of the cells for 9 days in G418 medium; typically, 80–90% of total cells were G418 resistant. The number of 6-TG$^R$ mutants was corrected for plating efficiency and for G418 resistance. All animal procedures were carried out in accordance with guidelines of the Canadian Council on Animal Care and the Animals for Research Act RSO-1990

### *Karyotype analysis*

Metaphase cells were prepared by addition of 0.1 $\mu$g ml$^{-1}$ colcemid 4 h before harvest. Cells were allowed to swell in 0.075 M potassium chloride for 15–20 min, then fixed in three changes of methanol–acetic acid (3:1, v/v) and dropped onto microscope slides. Identification of X chromosomal material in these cells was accomplished by hybridisation with a biotinylated X chromosome-specific composite DNA probe (Breneman *et al.*, 1994). Hybridisation and detection of bound probe was accomplished with two layers of avidin–FITC as described (Breneman *et al.*, 1994). Representative cells were photographed with Kodak Etkachrome 400 film on a Zeiss Axiophot photo microscope.

### Results

### *Karyotype analysis of MC1A derivatives*

MC1A was originally derived from a tumour arising in a male mouse injected with a carcinogen, methylcholanthrene (Kadhim and Rees, 1984; Kadhim *et al.*, 1987). It was anticipated that it would be aneuploid but the number of X chromosomes was uncertain. Since it had proven to be rather difficult to obtain HPRT$^-$ (6-TG$^R$) mutants of MC1A-C1 by MNU treatment or cobalt-60 $\gamma$-rays, this suggested that more than one functional copy of the X-linked *hprt* gene was present. Karyotype analysis, including the use of new X-specific hybridisation probes (Breneman *et al.*, 1995), was carried out (Table I, Figure 1). All lines were found to be hypotetraploid with about three X chromosomes plus other regions detected by the fluorescent probe, consistent with the notion of multiple copies of the *hprt* gene in MC1A.

### *Establishing conditions for growth in 6-TG to minimise metabolic cooperation*

Mutant cells lacking *hprt* (normally resistant to 6-TG) can still be killed by 'metabolic cooperation', i.e. passive acquisition of 6-TG nucleotide through gap junctions from neighbouring cells that contain active enzyme (Trosko and Chang, 1984). A reconstruction experiment was carried out to determine the cell density at which this effect could be minimised for our cell lines. One hundred 6-TG$^R$ MC-TGR17 cells were seeded onto 10 cm tissue culture dishes with increasing numbers of 6-TG$^S$ MC-TGS17-51 cells. The cells were grown in 6-TG for 14 days, following which 6-TG$^R$ colonies were

**Table I** Karyotype of MC1A-C1 and its derivatives

| Cell line | Total number of chromosomes | Number of X chromosomes | Number of other painted chromosomes |
|---|---|---|---|
| MC1A-C1 ($n = 19$) | 71.3 ± 14.3 | 2.8 ± 0.6 | 3.4 ± 1.4 |
| MC-TGR17 ($n = 39$) | 65.6 ± 5.0 | 2.8 ± 0.6 | 3.2 ± 0.8 |
| MC-TGS17-51 ($n = 20$) | 93.5 ± 11.1** | 4.7 ± 0.9** | 3.9 ± 1.1 |

Results represent means ± standard deviations. The differences in number of total chromosomes and X chromosomes are both statistically significant when MC-TGS17-51 was compared with the other two strains (**$P < 0.001$, ANOVA, Bonferroni post test). Other details given in Materials and methods.

**Figure 1** Detection of X chromosome-specific sequences by fluorescence *in situ* hybridisation in MC-TGR cells. Details as described in Materials and methods. The large arrows locate multiple X-chromosomes. Small arrows point to examples of interstitial and centromeric X-specific sequences.

counted. The recovery of 6-TG$^R$ colonies was unchanged in the presence of up to $1 \times 10^5$ 6-TG$^S$ cells per 10 cm dish. Above this cell number, there was a clear decrease in recovery of 6-TG$^R$ colonies such that, at $1 \times 10^6$ cells, very few colonies were recovered (data not shown). All experiments were therefore conducted at $1 \times 10^5$ cells per 10 cm dish.

*Spontaneous mutation frequency and growth rates of different HAT$^R$ clones*

The frequency of spontaneous mutants constitutes a background which affects the sensitivity of detection of induced mutants. To obtain cell lines with the lowest background possible, we screened 54 independently arising MC-TGS (HAT$^R$, 6-TG$^S$) clones to identify those with a low rate of spontaneous loss of the HPRT function. These clones were first grown in HAT medium (to eliminate any accumulated 6-TG$^R$ spontaneous mutants) and then grown in non-selective medium for up to 28 days, following which 6-TG$^R$ colonies were scored. Three clones were selected and analysed in greater detail for rate of spontaneous loss of HPRT function. At times up to 28 days following removal of HAT, clones were challenged with 6-TG (Figure 2a). In all cases, there was an increase in the number of 6-TG$^R$ colonies seen as a function of time in non-selective medium. Clone MC-TGS17-51 appeared to be the most stable. Its spontaneous mutation frequency was estimated to be $7.1 \times 10^{-6} \pm 0.2 \times 10^{-6}$ per day in one set of experiments (Figure 2a) and $8.2 \times 10^{-6} \pm 0.8 \times 10^{-6}$ per day in a second set of experiments (Figure 2b). The equivalent rate for the two other clones (17-1 and 17-25) ranged from 10 to $16 \times 10^{-6}$ per day. The growth rates of the three MC-TGS clones were not detectably different from parental MC1A-C1; doubling times were about 20 h (data not shown). Three G418-resistant subclones derived from MC-TGS17-51, namely, MN-5, MN-11 and MN-12, were also tested (Figure 2b). The spontaneous mutation frequency of MN-11 was estimated to be $5.1 \times 10^{-6} \pm 0.2 \times 10^{-6}$ per day. All three MN clones had a significantly shorter doubling time (15–16 h) than the parental clone (data not shown). When corrected for differences in doubling times, the rate of mutation was estimated to be $6.4 \times 10^{-6}$ cells per generation for MC-TGS17-51 and $3.3 \times 10^{-6}$ cells per generation for MN-11. The average growth rate of a pool of five spontaneously arising 6-TG$^R$ mutant clones was not detectably different from parental MN-11 cells, as assessed by a mixing experiment in which 6-TG$^R$ mutants were grown in the presence of MN-11 cells (data not shown).

*Radiation-induced 6-TG$^R$ mutants*

Our objective was to develop a cell line which could detect loss of *hprt* gene function with high sensitivity, that is, have a high ratio of induced to spontaneous mutations. We screened three MC-TGS17 clones to assess their sensitivity to mutation induction by cobalt-60 γ-radiation. Within experimental error, the number of induced mutations was similar (data not shown). MC-TGS17-51 was chosen because of its stability at the *hprt* locus. MC-TGS17-51, a G418$^R$ derivative (MN-11) and parental MC1A-C1 cells were compared in terms of sensitivity to induction of 6-TG$^R$ mutants by cobalt-60 γ-rays (Figure 3). Dose-dependent increases in the number of 6-TG$^R$ colonies were seen in the case of MC-TGS17-51 and MN-11, while virtually no 6-TG$^R$ colonies were observed in the parental MC1A-C1 cells. In fact, no 6-TG$^R$ colonies were detected in three independent experiments involving a total of $3.4 \times 10^6$ unirradiated MC1A-C1 cells and $1.5 \times 10^7$ irradiated (0.5–5 Gy) cells. The proposed explanation is that multiple functional copies of the X-linked *hprt* gene are present in MC1A-C1 cells (Table I and Figure 1). The induced levels of 6-TG$^R$ colonies in MC-TGS17-51 and MN-11 were 451 and 300 per $10^6$ viable cells per Gy respectively. At 5 Gy, we estimate that the induction of 6-TG$^R$ colonies in the derived clones was at least 1000-fold greater than in the

**Figure 2** Spontaneous generation of 6-TG$^R$ mutants. (**a**) Three different MC-TGS clones were grown in non-selective medium for the indicated periods of time, then challenged with 6-TG as described in Materials and methods. Error bars represent the s.e.m. of the average 3–9 replicate plates. (**b**) MN subclones, compared with MC-TGS17-51. Note difference in scale for parts (**a**) and (**b**). Error bars represent the s.e.m. of the average of 3–6 experiments, each involving 8–10 replicate plates.

**Figure 3** Generation of 6-TG$^R$ mutants in MC1A-C1 and various derivatives by cobalt-60 $\gamma$-rays. ●, MC-TGS17-51; ▲, MN-11; ■, parental MC1A-C1. Error bars represent the s.e.m. of the average of 3–5 experiments, each involving 3–10 replicate plates. Other details as described in Materials and methods.

MC1A-C1 parental line. Sensitivity to cell killing by ionising radiation was similar for the three lines, $D_{37}$ = 3.5 Gy for MC-TGS17-51 and MN-11 and 2.8 Gy for MC1A-C1 (data not shown).

*Detection of HPRT$^-$ mutants arising in tumours*

The primary use of the experimental model was to determine whether the frequency of HPRT$^-$ mutants is altered as a result of *in vivo* growth in subcutaneous tumours. Before injection, three MC-TGS17 clones with different spontaneous mutation frequencies *in vitro* were cultured in HAT-medium to remove pre-existing 6-TG$^R$ mutants. Once tumours reached about 1 cm in size, they were collected and cells were plated in the presence of 6-TG to detect mutants that had arisen during tumour growth. The mutation frequency of these cells and cells grown *in vitro* under non-selective conditions for an equivalent period of time is shown in Figure 4. There was a trend towards higher mutation frequency in cells derived from tumours as compared with cultured cells. Cells derived from MC-TGS17-36 tumours had a statistically significantly higher mutation frequency than cells from MC-TGS17-51 tumours. To explore in more detail the relationship between *in vitro* and *in vivo* mutation frequencies, MN-11 cells, which have a low background of spontaneous mutation and a selectable marker to allow distinguishing tumour from host cells, were used (Figure 5). The mutation frequencies of cells from 16 tumours (12–17 days of *in vivo* growth) and 12 independent cultures were compared. The differences in frequencies were highly significant ($P$<0.0001). The *in vivo* frequency was 3.4 times higher than the *in vitro* frequency. Tumour data were also examined as a function of time to reach 1 cm diameter; no statistically significant differences were detected (Figure 5, inset).

### Discussion

Genomic instability is a commonly observed feature of tumours (Nowell, 1982, 1991; Bringuier *et al.*, 1993; Nielsen *et al.*, 1993). Factors *intrinsic* to tumour cells (such as mutated forms of p53) may predispose these cells to further genomic instability (Weinert and Lydall, 1993). A second (not mutually exclusive) possibility is that instability is due to *extrinsic* factors in the tumour environment, such as reactive oxygen species or nitric oxide generated by phagocytic cells. To explore the latter hypothesis, we have developed a new *in vitro*/*in vivo* model system. A mouse tumour known to be infiltrated with macrophages and granulocytes, and which is readily transplantable in syngeneic animals, was chosen as a starting point (Kadhim and Rees, 1984; Kadhim *et al.*, 1987). A tissue culture line was established from the tumour, verified to be tumorigenic, and then genetically altered to

**Figure 4** Detection of 6-TG$^R$ mutants arising *in vivo* in different MC-TGS tumours. ■, The mutation frequency of cells grown *in vitro* under non-selective conditions for 14–21 days before being challenged with 6-TG. The mean ± s.e.m. of three replicate dishes in a single experiment is shown. ▨, The mean mutation frequency of cells recovered from different MC-TGS tumours. The error bars indicate the s.e.m. of four tumours (for 17–51) and five tumours (for 17–25 and 17–36). The tumour data were compared by one-way ANOVA and a post Tukey–Kramer test, which showed a statistically significant difference between 17–51 and 17–36 ($P<0.01$).

**Figure 5** Detection of 6-TG$^R$ mutants arising *in vivo* in MN-11 tumours. ■, The mean mutation frequency of MN-11 cells grown *in vitro* under non-selective conditions for 12–17 days before being challenged with 6-TG. The error bar indicates the s.e.m. of 12 independent experiments, each involving 6–7 replicate dishes of cells. ▨, The mean mutation frequency of cells recovered from subcutaneous tumours at 12–17 days after inoculation. The error bar indicates the s.e.m. of 16 separate tumours, each analysed using six replicate dishes. Mutation frequency is corrected for the percentage of cells (80–90%) which were G418 resistant. Mutations in cultured and tumour cells were compared by an unpaired *t*-test; two-tailed $P<0.0001$. Inset, the pooled tumour data in the main panel, shown by time of *in vivo* growth. The error bars indicate the s.e.m., where the number of tumours is indicated in parentheses. No statistically significant differences were detected.

allow more sensitive detection of mutagenic events. We chose the *hprt* gene as a surrogate for measuring cancer-related genetic changes since loss of function of this gene can be readily scored. A potential disadvantage of X-linked genes, such as *hprt*, is that mutations resulting in multilocus deletions (e.g. those caused by ionising radiation) may be non-viable if neighbouring essential genes are also lost. This biases the assay against such events (Evans *et al.*, 1986; Bradley *et al.*, 1988; Sankaranarayanan, 1991; Schwartz *et al.*, 1991; Zhou *et al.*, 1993). To overcome this problem, many workers have introduced an exogenous marker gene (Ashman and Davidson, 1985; Ashman, 1989; Ikehata *et al.*, 1989; Tindall and Stankowski, Jr. 1989; Kimura *et al.*, 1993; Lichtenauer-Kaligis *et al.*, 1993). Our difficulty in obtaining HPRT$^-$ mutants of MC1A-C1 and the subsequent detection of three copies of the X chromosome suggested a different strategy. Since it appeared that MC1A-C1 had more than one active X chromosome and more than one active *hprt* gene, we chose to create a *hprt* heterozygote, as has been carried out at the *tk* and *aprt* loci (Liber and Thilly, 1982; Sebastio *et al.*, 1985). This was done by screening for revertants to HAT$^R$. Clone MC-TGS17-51 and its derivatives were all very similar with respect to stability of *hprt* (Figure 2b), with spontaneous mutant frequencies estimated to be $<7.0\times10^{-6}$ mutants per generation, corrected for plating efficiency. There was a very large gain in sensitivity (>1000-fold) for detection of radiation-induced *hprt* mutations, compared with the parental line that is presumed to express multiple copies of *hprt*. The frequency of *hprt* mutations induced by cobalt-60 γ-rays in the cell lines we describe ($300-450\times10^{-6}$ per Gy) is within the range of that reported for other heterozygous marker genes (*tk*, *aprt* and *gpt*) in mouse and Chinese hamster lines (Evans *et al.*, 1986; Bradley *et al.*, 1988; Ikehata *et al.*, 1989; Schwartz *et al.*, 1991).

Our tumour model was developed to study factors in the tumour environment that cause mutagenic or clastogenic events. Clone MN-11, which exhibited the lowest *in vitro* rate of spontaneous gene loss, was used to test whether the *in vivo* environment was 'mutagenic', that is, whether an increase in mutation frequency could be detected compared with *in vitro* growth conditions. A highly statistically significant 3.4-fold increase in mutant frequency was observed. It is difficult to make precise estimates of the growth rate of cells *in vivo*, since tumours contain a mixture of growing, non-growing and dying cells. Thus, estimates of *in vivo* mutation *frequency* (mutants per total number of viable cells) not mutation *rate* (mutants per cell generation) are presented. It is worth noting that the doubling time of MN-11 cells *in vitro* is very rapid (16 h); hence, it is improbable that the observed increase in mutation frequency can be explained solely by a greater number of cell divisions *in vivo* compared with *in vitro*. A large variance in mutant frequency among different tumours was seen, compared with the small variance observed in replicates of a single tumour cell suspension. This suggests a Luria–Delbrück type of fluctuation (Kendal and Frost, 1988). To the best of our knowledge, these data are the first to directly demonstrate that factors in the tumour environment may cause mutations at the *hprt* locus in a murine syngeneic tumour model.

Most solid tumours, including human tumours, are infiltrated with inflammatory cells, and it is well known that a variety of inflammatory conditions such as ulcerative colitis predispose to malignancy (Templeton, 1975; Camisa, 1984; Yamada and Grisham, 1991; Babbs, 1992; Frenkel, 1992;

Levin, 1992). While recent attention has been paid to *endogenous* changes in tumour cells such as p53 and possibly p16 mutations that can predispose cells to genomic instability (Livingstone *et al.*, 1992; Tlsty *et al.*, 1992; Kamb *et al.*, 1994), the new experimental system we have described will allow us to study whether *exogenous* factors in the tumour environment such as pH, oxyradicals, nitric oxide, and others that may vary with the number of infiltrating inflammatory cells and the necrotic state of the tumour (Yamashina *et al.*, 1986; Dobrowsky *et al.*, 1991; Mareel *et al.*, 1991; Bennett *et al.*, 1993; Ohshima and Bartsch, 1994; Rosin *et al.*, 1994*a,b*) are also important in genomic instability.

**Abbreviations**

*aprt*, adenine phosphoribosyltransferase gene; *gpt*, bacterial xanthine guanine phosphoribosyltransferase gene; HAT, hypoxanthine, aminopterin and thymidine; $HAT^R$, cells resistant to HAT; *hprt*, hypoxanthine-guanine phosphoribosyltransferase gene; $HPRT^-$, cells lacking functional *hprt*; *neo*, bacterial neomycin gene; *pgk*, phosphoglycerate kinase; 6-TG, 6-thioguanine; $6\text{-}TG^R$, cells resistant to 6-TG; *tk*, thymidine kinase gene.

**Acknowledgements**

This work was supported by grants from the National Cancer Institute of Canada and the Medical Research Council of Canada to HCB. HCB is a Career Scientist of the Ontario Cancer Treatment and Research Foundation. This work was performed in part under the auspices of the US DOE by the Lawrence Livermore National Laboratory under Contract No. W-7405-ENG-48. We thank Dr David Blakey for his helpful comments during preparation of the manuscript.

**References**

ASHMAN CR. (1989). Retroviral shuttle vectors as a tool for the study of mutational specificity (base substitution deletion mutational hotspot). *Mutat. Res.*, **220**, 143-149.

ASHMAN CR AND DAVIDSON RL. (1985). High spontaneous mutation frequency of BPV shuttle vector. *Somat. Cell Mol. Genet.*, **11**, 499-504.

BABBS CF. (1992). Oxygen radicals in ulcerative colitis. *Free Radical Biol. Med.*, **13**, 169-181.

BENNETT SAL, LEITE LCC AND BIRNBOIM HC. (1993). Platelet activating factor, an endogenous mediator of inflammation, induces phenotypic transformation of rat embryo cells. *Carcinogenesis*, **14**, 1289-1296.

BENNETT SAL, CHEN J-H AND BIRNBOIM HC. (1994). Recovery of a rare clone from a population of unstable retroviral vector-expressing mammalian cells using a new RNA extraction and slot-blot protocol. *J. Virol. Methods*, **50**, 245-255.

BIRNBOIM HC. (1983). Importance of DNA strand-break damage in tumor promotion. In *Radioprotectors and Anticarcinogens*, Nygaard, OF and Simic MG (eds.) pp. 539-556. Academic Press: New York.

BRADLEY WEC, BELOUCHI A AND MESSING K. (1988). The *aprt* heterozygote hemizygote system for screening mutagenic agents allows detection of large deletions. *Mutat. Res.*, **199**, 131-138.

BRENEMAN JW, SWIGER RR, RAMSEY MJ, LEE DA, MINKLER JL, EVELETH JG, LANGLOIS RA AND TUCKER JD. (1995). The development of painting probes for dualcolor and multiple chromosome analysis in the mouse. *Cytogenet. Cell. Genet.*, **68**, 197-202.

BRINGUIER PP, BOUVIER R, BERGER N, PIATON E, REVILLARD JP, PERRIN P AND DEVONEC M. (1993). DNA ploidy status and DNA content instability within single tumors in renal cell carcinoma. *Cytometry*, **14**, 559-564.

CAMISA C. (1984). Squamous cell carcinoma arising in acne conglobata. *Cutis*, **33**, 185-190.

COLE J AND SKOPEK TR. (1994). International Commission for Protection Against Environmental Mutagens and Carcinogens. Working paper no. 3. Somatic mutant frequency, mutation rates and mutational spectra in the human population *in vivo*. *Mutat. Res.*, **304**, 33-105.

DOBROWSKY E, NEWELL K AND TANNOCK IF. (1991). The potential of lactate and succinate to kill nutrient deprived tumor cells by intracellular acidification. *Int. J. Radiat. Oncol. Biol. Phys.*, **20**, 275-279.

EMERIT I AND CERUTTI P. (1983). Clastogenic action of tumor promoter phorbol-12-myristate-13 acetate in mixed human leukocyte cultures. *Carcinogenesis*, **4**, 1313-1316.

EVANS HH, MENCL J, HORNG M-F, RICANATI M, SANCHEZ C AND HOZIER J. (1986). Locus specificity in the mutability of L5178Y mouse lymphoma cells: the role of multilocus lesions. *Proc. Natl Acad. Sci. USA.*, **83**, 4379-4383.

FRENKEL K. (1992). Carcinogen-mediated oxidant formation and oxidative DNA damage. *Pharmacol. Ther.*, **53**, 127-166.

FUSCOE JC, OCKEY CH AND FOX M. (1986). Molecular analysis of X-ray-induced mutants at the HPRT locus in V79 Chinese hamster cells. *Int. J. Radiat. Biol.*, **49**, 1011-1020.

HEPPNER GH, LOVELESS SE, MILLER FR, MAHONEY KH AND FULTON AM. (1984). Mammary tumor heterogeneity. In *Cancer Invasion and Metastasis: Biologic and Therapeutic Aspects*, Nicolson, GL and Milas L. (eds.) pp. 209-221. Raven Press: New York.

IKEHATA H, KIMURA H AND KATO T. (1989). Shuttle vector system for the analysis of mutational events in mammalian chromosomal DNA. *Mutat. Res.*, **210**, 237-247.

KADHIM SA, BURNS BF AND BIRNBOIM HC. (1987). In vivo induction of tumor variants by phorbol 12-myristate 13-acetate. *Cancer Lett.*, **38**, 209-214.

KADHIM SA AND REES RC. (1984). Enhancement of tumor growth in mice: evidence for the involvement of host macrophages. *Cell. Immunol.*, **87**, 259-269.

KAMB A, GRUIS NA, WEAVER-FELDHAUS J, LIU Q, HARSHMAN K, TAVTIGIAN SV, STOCKERT E, DAY RSI, JOHNSON BE AND SKOLNICK MH. (1994). A cell cycle regulator potentially involved in genesis of many tumor types. *Science*, **264**, 436-440.

KENDAL WS AND FROST P. (1988). Pitfalls and practice of Luria-Delbruck fluctuation analysis: a review. *Cancer Res.*, **48**, 1060-1065.

KIMURA H, HIGUCHI H, IYEHARA-OGAWA H AND KATO T. (1993). Sequence analysis of X-ray-induced mutations occurring in a cDNA of the human hprt gene integrated into mammalian chromosomal DNA. *Radiat. Res.*, **134**, 202-208.

KÖBERLE B AND SPEIT G. (1991). Molecular characterization of mutations at the *hprt* locus in V79 Chinese hamster cells induced by bleomycin in the presence of inhibitors of DNA repair. *Mutat. Res.*, **249**, 161-167.

LEACH FS, NICOLAIDES NC, PAPADOPOULOS N, LIU B, JEN J, PARSONS R, PELTOMäKI P, SISTONEN P, AALTONEN LA, NYSTRÖM-LAHTI M, GUAN XY, ZHANG J, MELTZER PS, YU J-W, KAO F-T, CHEN DJ, CEROSALETTI KM, FOURNIER REK, TODD S, LEWIS T, LEACH RJ, NAYLOR SL, WEISSENBACH J, MECKLIN J-P, JäRVINEN H, PETERSEN GM, HAMILTON SR, GREEN J, JASS J, WATSON P, LYNCH HT, TRENT JM, DE LA CHAPELLE A, KINZLER KW AND VOGELSTEIN B. (1993). Mutations of a *mut*S homolog in hereditary nonpolyposis colorectal cancer. *Cell*, **75**, 1215-1225.

LEVIN B. (1992). Ulcerative colitis and colon cancer: biology and surveillance. *J. Cell Biochem.*, **16G**, (suppl.) 47-50.

LIBER HL AND THILLY WG. (1982). Mutation assay at the thymidine kinase locus in diploid human lymphoblasts. *Mutat. Res.*, **94**, 467-485.

LICHTENAUER-KALIGIS EGR, THIJSSEN J, DEN DULK H, VAN DE PUTTE P, TASSERON-DE JONG JG AND GIPHART-GASSLER M. (1993). Genome wide spontaneous mutation in human cells determined by the spectrum of mutations in hprt cDNA genes. *Mutagenesis*, **8**, 207-220.

LIVINGSTONE LR, WHITE A, SPROUSE J, LIVANOS E, JACKS T AND TLSTY TD. (1992). Altered cell cycle arrest and gene amplification potential accompany loss of wild-type p53. *Cell*, **70**, 923-935.

LOEB LA. (1991). Mutator phenotype may be required for multistage carcinogenesis. *Cancer Res.*, **51**, 3075-3079.

MAREEL MM, DE BAETSELIER P AND VAN ROY FM. (1991). *Mechanisms of Invasion and Metastasis*, pp. 221-266. CRC Press: Boca Raton, FL.

MORITA H, UMEDA M AND OGAWA HI. (1991). Mutagenicity of various chemicals including nickel and cobalt compounds in cultured mouse FM3A cells. *Mutat. Res.*, **261**, 131-137.

NASSI-CALÒ L, MELLO FILHO AC AND MENEGHINI R. (1989). *o*-Phenanthroline protects mammalian cells from hydrogen peroxide-induced gene mutation and morphological transformation. *Carcinogenesis*, **10**, 1055-1057.

NICKLAS JA, O'NEILL JP, HUNTER TC, FALTA MT, LIPPERT MJ, JACOBSON-KRAM D, WILLIAMS JR AND ALBERTINI RJ. (1991). In vivo ionizing irradiations produce deletions in the hprt gene of human T-lymphocytes. *Mutat. Res.*, **250**, 383-396.

NIELSEN JL, WALSH JT, DEGEN DR, DRABEK SM, MCGILL JR AND VON HOFF DD. (1993). Evidence of gene amplification in the form of double minute chromosomes is frequently observed in lung cancer. *Cancer Genet. Cytogenet.*, **65**, 120-124.

NOWELL PC. (1982). Genetic instability in cancer cells: Relationship to tumor cell heterogeneity. In *Tumor Cell Heterogeneity: Origins and Implications*, Owens AH, Jr, Coffey DS and Baylin SB (eds.) pp. 351-365. Academic Press: New York.

NOWELL PC. (1991). Genetic instability and tumor development. *Basic Life Sci.*, **57**, 221-228.

OSHIMA H AND BARTSCH H. (1994). Chronic infections and inflammatory processes as cancer risk factors: Possible role of nitric oxide in carcinogenesis. *Mutat. Res. Fundam. Mol. Mech. Mutagen.*, **305**, 253-264.

PARSONS R, LI G-M, LONGLEY MJ, FANG W-H, PAPADOPOULOS N, JEN J, DE LA CHAPELLE A, KINZLER KW, VOGELSTEIN B AND MODRICH P. (1993). Hypermutability and mismatch repair deficiency in RER$^+$ tumour cells. *Cell*, **75**, 1227-1236.

ROSIN MP, ANWAR WA AND WARD AJ. (1994a). Inflammation, chromosomal instability, and cancer: The schistosomiasis model. *Cancer Res.*, **54**, Suppl. 1929s-1933s.

ROSIN MP, SAAD EL DIN ZAKI S, WARD AJ AND ANWAR WA. (1994b). Involvement of inflammatory reactions and elevated cell proliferation in the development of bladder cancer in schistosomiasis patients. *Mutat. Res. Fundam. Mol. Mech. Mutagen.*, **305**, 283-292.

SANKARANARAYANAN K. (1991). Ionizing radiation and genetic risks. III. Nature of spontaneous and radiation-induced mutations in mammalian in vitro systems and mechanisms of induction of mutations by radiation. *Mutat. Res.*, **258**, 7597.

SCHWARTZ JL, ASHMAN CR, ATCHER RW, SEDITA BA, SHADLEY JD, TANG J, WHITLOCK JL AND ROTMENSCH J. (1991). Differential locus sensitivity to mutation induction by ionizing radiations of different LETs in Chinese hamster ovary K1 cells. *Carcinogenesis*, **12**, 1721-1726.

SEBASTIO G, RICCIO A, VERDE P, SCARPATO N AND BLASI F. (1985). BamHI RFLP linked to the human urokinase gene. *Nucleic Acids Res.*, **13**, 5404.

STOUT JT AND CASKEY CT. (1985). HPRT: Gene structure, expression, and mutation. *Ann. Rev. Genet.*, **19**, 127-148.

TEMPLETON AC. (1975). Acquired diseases. In *Persons at High Risk of Cancer: an Approach to Cancer Etiology and Control*, Fraumeni JF, Jr. (ed.) pp. 69-84. Academic Press: New York.

TINDALL KR AND STANKOWSKI LF JR. (1989). Molecular analysis of spontaneous mutations at the *gpt* locus in Chinese hamster ovary (AS52) cells. *Mutat. Res.*, **220**, 241-253.

TLSTY TD, WHITE A AND SANCHEZ J. (1992). Suppression of gene amplification in human cell hybrids. *Science*, **255**, 1425-1427.

TROLL W AND WIESNER R. (1985). The role of oxygen radicals as a possible mechanism of tumor promotion. *Ann. Rev. Pharmacol. Toxicol.*, **25**, 509-528.

TROSKO JE AND CHANG C-C. (1984). Role of intercellular communication in tumor promotion. In *Mechanisms of Tumor Promotion. Vol. IV. Cellular Responses to Tumor Promoters*, Slaga TJ. (ed.) pp. 119-145. CRC Press: Boca Raton, FL.

VRIELING H, SIMONS JW, ARWERT F, NATARAJAN AT AND VAN ZEELAND AA. (1985). Mutations induced by X-rays at the HPRT locus in cultured Chinese hamster cells are mostly large deletions. *Mutat. Res.*, **144**, 281-286.

VRIELING H, NIERICKER MJ, SIMONS JW AND VAN ZEELAND AA. (1988). Molecular analysis of mutations induced by *N*-ethyl-*N*-nitrosourea at the HPRT locus in mouse lymphoma cells. *Mutat. Res.*, **198**, 99-106.

WEINERT T AND LYDALL D. (1993). Cell cycle checkpoints, genetic instability and cancer. *Semin. Cancer Biol.*, **4**, 129-140.

WEITZMAN SA AND WEITBERG AB. (1985). Phagocytes as carcinogens: Malignant transformation produced by human neutrophils. *Science*, **227**, 1231-1233.

YAMADA T AND GRISHAM MB. (1991). Role of neutrophil-derived oxidants in the pathogenesis of intestinal inflammation. *Klin. Wochenschr*, **69**, 988-994.

YAMASHINA K, MILLER BE AND HEPPNER GH. (1986). Macrophage-mediated induction of drug-resistant variants in a mouse mammary tumor cell line. *Cancer Res.*, **46**, 2396-2401.

YUNIS JJ. (1983). The chromosomal basis of human neoplasia. *Science*, **221**, 227-236.

ZHOU PK, LIU XY, SUN WZ, ZHANG YP AND WEI K. (1993). Cultured mouse SR-1 cells exposed to low dose of gamma-rays become less susceptible to the induction of mutagenesis by radiation as well as bleomycin. *Mutagenesis*, **8**, 109-111.